# Neuronal Network Inactivity Potentiates Neuropeptide Release from Mouse Cortical Neurons

**DOI:** 10.1523/ENEURO.0555-24.2024

**Published:** 2025-03-25

**Authors:** Theresa Priebe, Aygul Subkhangulova, Ruud F. Toonen, Matthijs Verhage

**Affiliations:** ^1^Department of Functional Genomics, Center for Neurogenomics and Cognitive Research, Neurosciences Campus Amsterdam, Vrije Universiteit Amsterdam, Amsterdam 1081 HV, The Netherlands; ^2^Department of Human Genetics, Center for Neurogenomics and Cognitive Research, Neurosciences Campus Amsterdam, Amsterdam University Medical Centers, Amsterdam 1081 HV, The Netherlands

**Keywords:** dense core vesicles, neuropeptides, synaptic silencing

## Abstract

Neurons adapt to chronic activity changes by modifying synaptic properties, including neurotransmitter release. However, whether neuropeptide release via dense core vesicles (DCVs)—a distinct regulated secretory pathway—undergoes similar adaptation remains unclear. Here, we demonstrate that 24 h action potential blockade leads to significant DCV accumulation in primary mouse cortical neurons of both sexes. Reactivation with action potential trains induced enhanced Ca^2+^ influx and 700% more DCV exocytosis compared with control neurons. Notably, total DCV cargo protein levels were unchanged, while mRNA levels of corresponding genes were reduced. Blocking neurotransmitter release with Tetanus toxin induced DCV accumulation, similar to that induced by network silencing with TTX. Hence, chronic network silencing triggers increased DCV accumulation due to reduced exocytosis during silencing. These accumulated DCVs can be released upon reactivation resulting in a massive potentiation of DCV exocytosis, possibly contributing to homeostatic mechanisms.

## Significance Statement

This study addresses an unexplored area—how dense core vesicles (DCVs) exocytosis adapt to chronic changes in activity—and demonstrates accumulation of DCVs and a massive upregulation of DCV exocytosis in response to 24 h inactivity. The potentiation of neuropeptide release might contribute to homeostatic regulation of neuronal networks in the brain.

## Introduction

Neurons adapt to chronic changes in activity by adjusting synapse properties to maintain neuronal activity levels and to balance excitation and inhibition (Turrigiano and Nelson 2000). This process involves mechanisms to maintain neuronal activity levels and regulate circuit connectivity (Turrigiano 1999, 2008; Davis and Bezprozvanny 2001; Burrone and Murthy 2003; Perez-Otano and Ehlers 2005; Davis 2006; Marder and Goaillard 2006; Yu and Goda 2009), on both the presynaptic and postsynaptic sides (Rao and Craig 1997; Lissin et al., 1998; O'Brien et al., 1998; Murthy et al., 2001; Moulder et al., 2006; Muller et al., 2012; Orr et al., 2022). For instance, chronic inhibition of neuronal activity can lead to an increase in the number of synaptic vesicles (SVs) that are ready for release, as well as an increased probability of release upon nerve stimulation (Murthy et al., 2001; Muller et al., 2015). Conversely, increased neuronal activity can result in reduced presynaptic release at excitatory synapses (Moulder et al., 2004, 2006; Branco et al., 2008). These studies have focused on homeostatic regulation of fast synaptic signaling by means of classical small-molecule neurotransmitters (glutamate, GABA). However, most neurons also secrete neuropeptides and neurotrophic factors which typically have slower neuromodulatory functions and regulate important behaviors, such as sleep (Chemelli et al., 1999), addiction (Zachariou et al., 2003), social bonding (Nagasawa et al., 2015), and pain (Liu et al., 2023). Neuropeptides may activate many receiving neurons due to a longer extracellular half-life than classical transmitters. These are released from dense core vesicles (DCVs), which differ from SVs in terms of morphology, composition, biogenesis, and the stimulation required for exocytosis. DCV exocytosis requires stronger stimulation and higher cytoplasmic calcium compared with SV exocytosis (Verhage et al., 1991; Nurrish 2014; Persoon et al., 2018; Baginska et al., 2023). Despite the critical role of neuropeptides in brain function, the mechanisms regulating neuropeptide release following chronic inactivity are not well understood.

Early studies indicated that neuronal activity generally regulates the expression of neuropeptides (Carnahan and Nawa 1995; MacArthur and Eiden 1996; Bouwman et al., 2006). More recent studies demonstrated how brain-derived neurotrophic factor (Bdnf) and somatostatin (Sst) are upregulated following increased network activity (Schaukowitch et al., 2017). Additionally, certain DCV components, such as receptor tyrosine phosphatase-like protein (Ptprn/IA-2), a well-established transmembrane DCV protein in pancreatic cells (Wasmeier and Hutton 1996), are upregulated by activity suppression but downregulated during periods of sustained increase in network activity (Wasmeier and Hutton 1996; Schanzenbacher et al., 2016; Dorrbaum et al., 2020). Furthermore, DCVs accumulate in the presynaptic compartment following chronic synaptic silencing (Tao et al., 2018). Despite these findings, how the mechanisms of neuropeptide release and the total pool of DCVs are influenced by changes in network activity remains largely unknown.

This study aimed to investigate the effects of neuronal network silencing on DCV exocytosis, the levels of DCV cargo proteins, and the total DCV pool. We addressed this question using fluorescence-based assays which allow detection of DCV exocytosis at a single vesicle resolution. Our findings indicate that silencing cultured cortical neurons led to an increase in DCV numbers within neurites and potentiated DCV exocytosis upon reactivation of neuronal activity. While protein levels remained unchanged, the mRNA levels of neuropeptides decreased after tetrodotoxin (TTX)-mediated network silencing. Collectively, these results demonstrate a strong potentiation of DCV exocytosis following prolonged inactivity, which may contribute to homeostatic principles in the brain.

## Material and Methods

### Biosafety

All experimental procedures were performed according to the local guidelines of the VU University/VU University Medical Centre. For lentiviral work, we followed safety measures according to European legislation (ML-II, permit number: IG16-223-IIk).

### Plasmids and virus

Constructs were cloned into pLenti vectors containing the neuron-specific synapsin promoter (Naldini et al., 1996). NPY-pHluorin and TeNT-IRES-mCherry were described before (Farina et al., 2015; Persoon et al., 2018; Hoogstraaten et al., 2020). The genetic calcium indicator Soma-GCamp7f under human synapsin1 promotor (Addgene plasmid # 158759) was used for calcium imaging experiments.

### Laboratory animals, primary neuron cultures, and infection

All animal experiments were approved by the animal ethical committee of the VU University/VU University Medical Centre (license number: FGA 11-03 and AVD112002017824). Animals were housed and bred according to institutional and Dutch governmental guidelines and regulations. Wild-type C57BL/6 mouse neurons of both sexes were obtained from embryonic day 18 (E18) embryos, by cesarean section of pregnant mice.

Mouse cortices were dissected in Hanks balanced salt solution (Sigma), supplemented with 10 mM HEPES (Invitrogen) and were digested with 0.25% trypsin (Invitrogen) in Hanks + HEPES for 15 min at 37°C. Hippocampi were washed three times with Hanks + HEPES, once with DMEM complete [DMEM + GlutaMAX (Invitrogen), supplemented with 10% FCS (Invitrogen), 1% NEAA (Sigma) and 1% penicillin/streptomycin (Sigma)] and triturated with fire-polished Pasteur pipettes. Dissociated cells were spun down and resuspended in Neurobasal medium (Invitrogen) supplemented with 2% B-27 (Invitrogen), 1.8% HEPES, 0.25% GlutaMAX (Invitrogen), and 0.1% penicillin/streptomycin. Continental cultures were created by plating neurons at 30 K/well. Neurons were seeded on pregrown rat glia on 18 mm glass coverslips in 12-well plates (Mennerick et al., 1995; Wierda et al., 2007). For Western blots and RNA extraction cortices were washed and triturated and 350,000 neurons/well were plated on 6-well plates coated with a solution of 0.5*10−3% poly-ʟ-ornithine and 2.5 μg/ml laminin (Sigma). Primary cortical neurons were infected at days in vitro (DIV) 9–12 and imaged 6 d after infection and fixed and stained after TTX application described in results.

### Imaging

TTX (1 μM) was applied 24 h before imaging. Neurons were imaged in Tyrode's solution (in mM: 2 CaCl_2_, 2.5 KCl, 119 NaCl, 2 MgCl_2_, 30 glucose, 25 HEPES, pH 7.4). Glutamate receptor antagonists 6-cyano-7 nitroquinoxaline-2,3-dione (CNQX; 10 μM) and d,l-2-amino-5-phosphonovaleric acid (APV; 5 μM) were included in the Tyrode's solution during the experiments involving stimulation. Imaging was performed with a custom-built microscope containing an imaging microscope (AxioObserver.Z1, Zeiss), 561 and 488 nm lasers, polychrome V, appropriate filter sets, 40× oil objective (NA 1.3) and an EM-CCD camera (C9100-02; Hamamatsu, pixel size 200 nm). Images were acquired with AxioVision software (version 4.8, Zeiss). Electrical stimulation was performed with two parallel platinum electrodes placed around the neuron. Sixteen trains of 50 pulses at 50 Hz, interspaced by 0.5 s, were initiated by a Master-8 (AMPI) and a stimulus generator (A-385, World Precision Instruments) delivered the 1 ms pulses of 30 mA. Tyrode's with 50 mM NH4 + Cl (replacing 50 mM NaCl) was delivered by gravity flow through a capillary placed above the neuron. Experiments were performed at room temperature (21–25°C).

DCV exocytosis events were detected manually as sudden appearance of NPY-pHluorin–positive puncta using ImageJ. NPY-pHluorin events were considered a fusion event if the maximal fluorescence was at least twice the SD above noise. Custom-written MATLAB scripts were used to calculate the number and timing of fusion events. Kymographs were generated with ImageJ of a neurite stretch positive for GCaMP7f.

### SDS-PAGE WB analysis

To analyze protein levels, high-density neuronal cultures (400 K/well in a six-well plate) were lysed in Laemmli sample buffer. Lysates were heated at 95°C for 5 min, separated by SDS-PAGE using either home-made Tris-glycine or commercially available Mini-PROTEAN TGX Stain-Free gels (Bio-Rad 4568096) and transferred to nitrocellulose membrane (Bio-Rad 1620115) using a wet tank transfer method. Membranes were blocked in 5% milk powder dissolved in Tris-buffered saline containing 0.1% Tween 20 (TBS-T). Membranes were incubated with primary antibodies diluted in TBS-T on a shaking platform overnight at 4°C. Primary antibodies used in this study and their dilutions are listed in the Table 1. Horseradish peroxidase coupled secondary antibodies were applied at 1:10,000 dilutions for 1 h at room temperature. Chemiluminescence-based detection was performed using SuperSignal West Femto Maximum Sensitivity Substrate (Thermo Fisher Scientific 34095) on the Odyssey Fc imaging system (LI-COR Bioscience). Immunosignal of some proteins (IA-2, BDNF) was detected using a more sensitive SuperSignal West Atto Ultimate Sensitivity Substrate (Thermo Fisher Scientific A38555). Signal intensities of bands of interest were analyzed using Image Studio Lite Software and normalized to the intensity of a loading control (actin, tubulin, or GAPDH).

**Table 1.** Key resources table

Reagent type (species) or resource	Designation	Source or reference	Identifiers	Additional information
Genetic reagent (*Mus musculus*)	C57BL/6 J	Charles River	Strain code 631	
Genetic reagent (*Rattus norvegicus*)	Wistar (Crl:WI)	Charles River	Strain code: 003	Used for preparation of glia feeder layer
Antibody	Anti-NPY (rabbit polyclonal)	Cell Signaling	11,976	1:400
Antibody	Anti-VAMP2 (mouse monoclonal)	Synaptic Systems	104211	1:2,000
Antibody	Anti-actin (mouse monoclonal)	Merck	MAB1501	1:1,000
Antibody	Anti-MAP-2 (chicken polyclonal)	Abcam	ab5392	1:5,000
Antibody	Anti-IA-2 (PTPRN) (mouse monoclonal)	Merck	MABS469	1:100
Antibody	Anti-CHGB (rabbit polyclonal)	Synaptic Systems	259103	1:500
Antibody	Anti-BDNF (mouse monoclonal)	DSHB hybridoma product	BDNF #9 (supernatant)^1^	1:4
Antibody	Anti-GAPDH (rabbit polyclonal)	Elabscience	E-AB-40337	1:2,000
Recombinant DNA reagent	pLenti-Syn(pr)- TeNT-IRES- mCherry	Hoogstraaten et al. (2020)	-	
Recombinant DNA reagent	pLenti-Syn(pr)-pre-NPY-pHluorin		-	
Sequence-based reagent	qPCR primers are Listed in Table 2			
Peptide, recombinant protein	2.5% trypsin	Invitrogen	15090046	
Peptide, recombinant protein	Papain	Worthington Biochemical Corporation	LS003127	
Peptide, recombinant protein	Poly-ʟ-ornithine	Sigma	P4957	
Peptide, recombinant protein	Laminin	Sigma	L2020	
Peptide, recombinant protein	Rat tail collagen	BD Biosciences	354,236	
Peptide, recombinant protein	Poly-d-lysine	Sigma	P6407	
Commercial assay or kit	SensiFast cDNA Synthesis Kit	Meridian Bioscience	BIO-65054	
Commercial assay or kit	SensiFast SYBR Lo-ROX Kit	Meridian Bioscience	BIO-94020	
Software, algorithm	MATLAB	MathWorks	-	
Software, algorithm	Prism	GraphPad	-	
Software, algorithm	Fiji/ImageJ	NIH	-	

### Quantitative RT-PCR

RNA extraction from neuronal cultures was performed using the ISOLATE II RNA Micro Kit (Meridian Bioscience BIO-52073). cDNA was synthesized from purified RNA using SensiFast cDNA Synthesis Kit (Meridian Bioscience BIO-65054). qRT-PCRs were run on a QuantStudio 5 system (Thermo Fisher Scientific) using SensiFast SYBR Lo-ROX Kit (Meridian Bioscience BIO-94020). qRT-PCR primers (listed in Table 2) were validated for the efficiency by running qRT-PCRs on 10-fold serial dilutions of cDNA and for the specificity estimated from melting curves (Subkhangulova et al., 2023). Only primers with an efficiency of 90–105% were used for experiments. Fold changes (FC) in gene expression were determined using the cycle threshold (CT) comparative method (2−ddCT) using GAPDH as a reference gene. GAPDH CT values were not affected by the treatment applied. Statistical analysis of the data was performed on log-transformed data (logFC, ddCT).

**Table 2.** List of qRT-PCR primers used in this study

Gene	Forward primer sequence	Reverse primer sequence
*Bdnf* (all transcripts)	GGCTGACACTTTTGAGCACGTC	CTCCAAAGGCACTTGACTGCTG
*Vgf*	CTTTGACACCCTTATCCAAGGCG	GCTAATCCTTGCTGAAGCAGGC
*Pam*	AGTCGGATCGTGCAGTTCTCAC	ACTGGTTCAGGTGAGGCACAAG
*Ptprn*	TGGCAGGCTATGGAGTAGAGCT	CTTGACATCGGCTCCTCCAACA
*ChgB*	GACGAATTTCCCGATTTCTAC	CCAGTTCCTTTTTCTCTTCCG
*Scg2*	CAGGAAGAGGTGAGAGACAGCA	TGGAGGCATCCTCTGAGAGTTG
*Npy*	TACTCCGCTCTGCGACACTACA	GGCGTTTTCTGTGCTTTCCTTCA
*Cck*	GAGGTGGAATGAGGAAACAA	CAGATTTCACATTGGGGACT
*Sst*	TCTGGAAGACATTCACATCC	TTCTAATGCAGGGTCAAGTT
*Gapdh*	CATCACTGCCACCCAGAAGACTG	ATGCCAGTGAGCTTCCCGTTCAG

### Immunocytochemistry

TTX (1 μM) was applied between 4 and 48 h before fixation. Neurons were fixed at DIV9, DIV14/15, or DIV21 in freshly prepared 3.7% paraformaldehyde (EMS) for 10 min at room temperature, permeabilized with 0.1% Triton X-100 (Fisher Scientific) for 10 min and blocked with 0.1% Triton X-100 and 2% normal goat serum for 30 min. Incubation with primary and secondary antibodies was done at room temperature for 1–2 h. All solutions were in PBS (composition in mM: 137 NaCl, 2.7 KCl, 10 Na_2_HPO_4_, 1.8 KH_2_PO_4_, pH 7.4). Coverslips were mounted in Mowiol (Sigma).

Primary antibodies used for immunocytochemistry are listed in Table 1.

### Data analysis

DCV fusion events were analyzed using a semiautomated DCV-pHluorin plug-in previously described (Persoon et al., 2019; Moro et al., 2021a), and fusion events are defined as a fast increase in fluorescence intensity. For DCV fusion, 3 × 3 pixels ROIs were placed semiautomatically, using a custom-made script in ImageJ, on all pHluorin region that appeared during the electrical stimulation. Individual traces were validated using a custom-made MATLAB script; only regions that showed an increase in *F*/*F*0 ≥2 SD and a rise time of <1 s were recorded as positive fusion events, with *F*0 calculated by averaging the first 10 frames of the time-lapse recording. The total number of DCVs was calculated based on the NH4Cl (Tyrode's solution containing 50 mM NH4Cl) response of individual recording with a custom-made ImageJ algorithm. Because of the overlap of DCVs in individual puncta, the number of vesicles was corrected normalizing the puncta intensity by the mode of the first percentile of the intensity distribution per cell. To calculate the released fraction, the number of fusion events per neuron was divided by the total intracellular pool of DCVs.

Neuronal morphology, DCV marker intensity quantification, and maximum intensity projections of confocal images were analyzed with a custom-made ImageJ algorithm, dendrites and axons based on Ridge detection, and their length was calculated based on the skeleton analysis in ImageJ; DCVs were identified based on their intensity and dimension.

The MAP2 masking was used to quantify DCVs in a neurite mask of each neuron. For neuronal morphology and DCV quantification, maximum intensity projections of confocal images were analyzed with a custom-made ImageJ algorithm, dendrites based on Ridge detection and their length were calculated on the basis of the skeleton analysis in ImageJ, and DCVs were identified on the basis of their intensity and dimension.

### Code availability

The custom code used for data analysis in ImageJ during the current study is available in GitHub at https://github.com/alemoro.

### Statistical analyses

Normal distributions for all datasets were assessed first using Shapiro–Wilk normality tests. To compare two groups, we used an unpaired Student's *t* test in the case of normal distributed or Mann–Whitney U tests for nonparametric data (all other cases). Data is represented as average with standard error of the mean (SEM). To test more than two groups, we used one-way analysis of variance (ANOVA) followed by Dunnett's multiple-comparisons test to compare conditions. Statistical analyses were performed using MATLAB R2021b (MathWorks) and GraphPad Prism version 10.3.1 for Mac OS (GraphPad Software; www.graphpad.com).

## Results

### TTX-induced network silencing potentiates neuropeptide release

**Figure 1.**
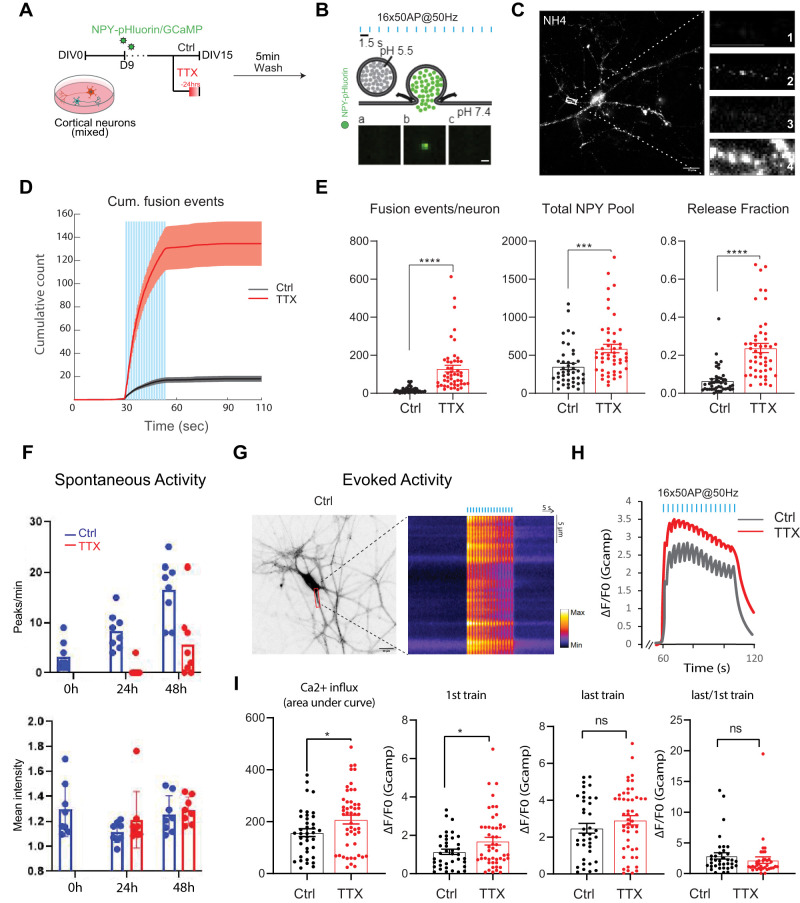
Neuronal network silencing with TTX results in increased DCV marker intensity in neurites and potentiated DCV exocytosis upon washout in mouse cortical neurons. ***A***, Experimental schematic manipulating network activity to induce synaptic scaling and measure effect on DCV fusion events (NPY-pHluorin) and calcium influx (GCaMP7f). Mouse cortical neurons were treated with sodium channel blocker (TTX, 1 µM) for 24 h before imaging. ***B***, NPY-pHluorin as optical reporter for DCV exocytosis, repetitive electrical stimulation (16 trains of 50 APs at 50 Hz) is represented by blue bars. ***C***, Exemplary image of a neuron during live cell imaging protocol. Full size image shows NPY-pHluorin puncta upon NH_4_^+^ perfusion for total DCV pool quantification (scale bar, 20 µm). Panels show a neurite before stimulation (1), during stimulation displaying fusion events (2), after stimulation (3), and the total number of NPY-pHluorin labeled DCVs upon NH_4_^+^ perfusion (4). Scale bar, 4 µm. ***D***, Cumulative plot of DCV fusion events in control (red), mean = 17,58, and TTX treated (black), mean = 129,4 neurons (after TTX washout). Shaded area represents SEM. Blue bars represent repetitive electrical stimulation (16 trains of 50 APs at 50 Hz). ***E***, Average DCV fusion events, total pool, and release fraction per cell in control (*n* = 43, *N* = 3) and TTX (*n* = 49, *N* = 3) treated neurons. Mann–Whitney *U* test: Fusion events: ****p = 3.887258× 10^−14^. Total Pool***p = 0.000195298. Release Fraction ****p = 8.73× 10^−11^. Bars represent mean + SEM. ***F***, Number of calcium induced fluorescence peaks in mass cultures of cortical neurons representing spontaneous network activity. Control (blue) and TTX (1 µM) incubated for 0, 24 or 48 h (red). *n* = 3. Neurons infected with GCaMP7f were imaged for 5 min in the presence of TTX (0 h), same neurons were imaged again for 5 min after 24 and 48 h. Mean intensity was measured placing a ROI around somas and background subtraction. ***G***, Example images of ctrl neuron expressing GCaMP7f (left) with Kymograph of intracellular calcium levels (right) upon repetitive electrical stimulation (blue bars). ***H***, Average normalized Δ*F* / *F*0 traces of intracellular calcium (GCaMP7f) of wild-type cortical neurons with (red) or without (gray) TTX, with indicated repetitive electrical stimulation (16 trains of 50 APs at 50 Hz) in blue bars. Mouse cortical neurons were treated with sodium channel blocker (TTX, 1 µM) 24 h before imaging. *N* = 3, *n* = 40–45. ***I***, Average of Ca2+ influx, 1st train, last train, and last/1st train per cell in control (*n* = 43, *N* = 3) and TTX (*n* = 49, *N* = 3) treated neurons. Mann–Whitney *U* test: Area under the curve: *p = 0.0319. 1st train: *p = 0.0365. Last train: ns, *p* = 0.2455. Last/1st train: ns, *p* = 0.3566. Bars represent mean + SEM. Mann–Whitney test, *p < 0.05, **p < 0.01, ***p < 0.001, ns *p* > 0.05. Dots represent individual neurons, *n* = number of neurons, *N* = number of independent experiments.

A previous study indicated that DCVs accumulate in the presynaptic compartment upon chronic network silencing (Tao et al., 2018). We asked if reactivation of the network results in exocytosis of these vesicles and increased release of cargo. Therefore, we treated mouse cortical neurons with 1 µM TTX for 24 h (Fig. 1*A*) and visualized exocytosis of DCVs after TTX washout at DIV15. For this, we used NPY-pHluorin, a pH-sensitive neuropeptide fusion reporter (de Wit et al., 2009; Farina et al., 2015; Persoon et al., 2018; Fig. 1*A*,*B*) that colocalizes with 90% of the endogenous DCV cargo Chromogranin B (ChgB). Importantly, expressing NPY-pHluorin in primary neurons does not alter the overall DCV pool or the staining intensity of individual DCV cargo (Persoon et al., 2018). NPY-pHluorin signals were measured upon 16 episodes of 50 pulses at 50 Hz, 5 min after TTX washout (Fig. 1*A–C*). TTX-treated neurons showed a sevenfold potentiated DCV exocytosis (Fig. 1*D*,*E*). The total pool of DCVS, which is visualized by NH4+ perfusion dequenching all pHluorin signal in the neuron, was increased (Fig. 1*C*,*E*). After correction for this increased total DCV pool, the release fraction (fusion events divided by the total pool; Fig. 1*E*) was still significantly increased in TTX-treated neurons.

In parallel, we used lentiviral expression of genetically encoded calcium indicator GCaMP7f to assess network activity (Fig. 1*A*,*F–I*). The first set of experiments were performed to control for the efficiency of TTX silencing. Neurons were imaged for 5 min, and calcium influx-induced fluorescence peaks were compared after 24 and 48 h of TTX application (Fig. 1*F*). Twenty-four hour after addition of TTX (1 μM), virtually no calcium spikes were registered. At 48 h after addition of the single dose of TTX, neurons slowly regained spontaneous activity, as evidenced by the reappearance of calcium spikes (Fig. 1*F*). This is likely due to degradation of TTX in culture medium, likely due to factors such as temperature or light exposure (Chen and Chung 2014). Therefore, in all our following experiments we replenished TTX in culture medium every 24 h. The second set of experiments (Fig. 1*G–I*) was performed to control for the efficiency of TTX washout. As expected, cultures incubated with TTX for 24 h show stimulation-evoked calcium spiking after washout with enhanced transient increases in intracellular [Ca^2+^], as shown previously (Zhao et al., 2011).

**Figure 2.**
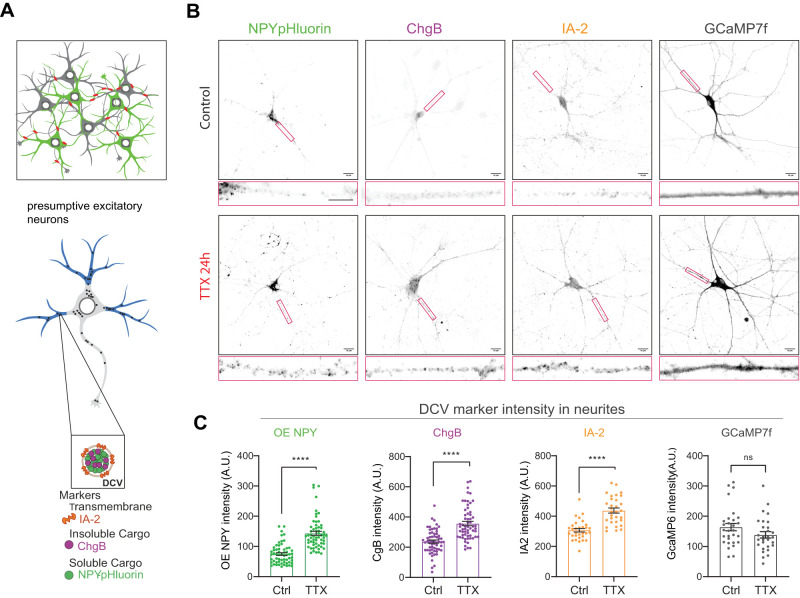
Blocking action potential-mediated synaptic activity leads to an increase in DCV marker intensities in neurites of mouse cortical neurons. ***A***, Experimental schematic. Top, Cortical neurons infected with either NPY-pHluorin or the genetic calcium indicator GCaMP7f (see also Fig. 1). Bottom, Scheme of a typical neuron stained for two different DCV markers and MAP-2 neurites indicated in blue. Fluorescence intensity of DCV markers was measured in neurites (blue). ***B***, Representative images of control or TTX-treated DIV15 neurons infected (DIV9) with NPY-pHluorin or the genetic calcium indicator GCaMP7f. Neurons were costained for ChgB or IA-2. Scale bar, 16 µm. Red boxes indicate position of zooms. Zoom scale bar 8µm. ***C***, Fluorescence marker intensity in neurites of mouse cortical neurons. NPY-pHluorin (*n* = 59, *N* = 3) ****p = 1.59× 10^−13^, ChgB (*n* = 60, *N* = 3) ****p = 1.36× 10^−09^, IA-2 (*n* = 31. *N* = 3) ****p = 9.10× 10^−07^, GCaMP7f (*n* = 29, *N* = 3) ns, *p* = 0.1005, control and TTX-treated neurons. Mann–Whitney *U* test: The data are presented as the mean ± SEM.

DCV accumulation was assessed by labeling neurites for DCV cargo. Cortical neurons infected (DIV9) with NPY-pHluorin or GCaMP7f were fixed 24 h after TTX application and costained, with endogenously expressed DCV cargo ChgB and transmembrane protein Ptprn/IA-2, with MAP-2 as a neurite mask (Fig. 2*A–C*). As expected, accumulation of DCVs was observed in MAP2 positive neurites (Fig. 2*B*,*C*). Quantification of fluorescence signal of different DCV markers in neurites revealed higher levels of all markers (NPY-pHluorin 1.8-fold, ChgB 1.4-fold, IA-2 1.4-fold) compared with Ctrl neurons (Fig. 2*C*), whereas GCaMP7f levels remained the same (Fig. 2*C*). These data confirm that after 24 h of network silencing, DCVs accumulate in neurites and reactivation results in an increased total DCV pool as well as DCV exocytosis and release efficacy.

### TTX-induced network silencing for 4 h accumulates DCVs in neurites

**Figure 3.**
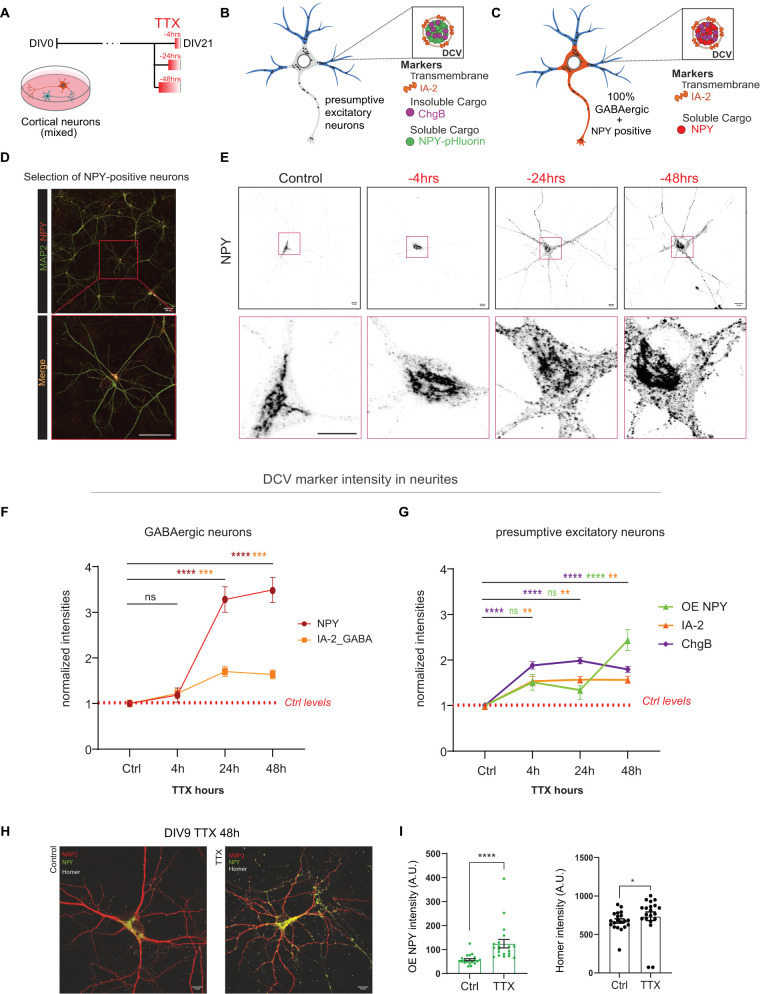
Blocking action potential-mediated synaptic activity leads to an increase in DCV marker intensities in GABAergic neurites of mouse cortical neurons. ***A***, Experimental schematic, manipulating network activity to induce synaptic scaling and measure effect on DCV numbers. Mouse cortical neurons were treated with sodium channel blocker (TTX, 1 µM) for 4, 24, or 48 h before fixation at DIV21. ***B***, ***C***, Scheme of a typical neuron stained for three different DCV markers. Fluorescence intensity of DCV markers was measured in neurites (blue). GABAergic neurons in red were stained for NPY and IA-2. ***D***, NPY-positive GABAergic neurons were manually selected on an array scan of the entire coverslip. Scale bar, 100 µm. ***E***, Representative composite confocal images of cortical neurons depicting NPY for every treated condition. Scale bars, 10 μm. Red squares indicate position of zooms of soma region showing NPY accumulation in Golgi area and increase in NPY-positive puncta outside Golgi after treatment with TTX. ***F***, Normalized quantified fluorescence markers NPY endogenous and IA-2 in neurites of mouse cortical neurons (*n* = 42–44, *N* = 3). NPY: ctrl versus 4 h *p* = 0.98, ctrl versus 24 h *p* < 0.0001, ctrl versus 48 h *p* < 0.0001. IA-2: ctrl versus 4 h *p* = 0.8073, ctrl versus 24 h *p* = 0.0002, ctrl versus 48 h *p* = 0.0007. The data are presented as the mean ± SEM. *p < 0.05; **p < 0.01; ***p < 0.001; and ****p < 0.0001. Ordinary one-way ANOVA. *n* = number of neurons, *N* = number of independent experiments. ***G***, Normalized quantified fluorescence markers in neurites of mouse cortical neurons. Different DCVs markers were labeled ChgB (*n* = 26, *N* = 3), IA-2 (*n* = 46, *N* = 3), and NPY-pHluorin (*n* = 26, *N* = 3). ChgB: ctrl versus 4 h *p* < 0.0001, ctrl versus 24 h *p* < 0.0001, ctrl versus 48 h *p* < 0.0001. IA-2: ctrl versus 4 h *p* = 0.0033, ctrl versus 24 h *p* = 0.0014, ctrl versus 48 h *p* = 0.0013. NPY-pHluorin: ctrl versus 4 h *p* = 0.1358, ctrl versus 24 h *p* = 0.5237, ctrl versus 48 h *p* < 0.0001. The data are presented as the mean ± SEM. *p < 0.05; **p < 0.01; ***p < 0.001; and ****p < 0.0001. Ordinary one-way ANOVA. *n* = number of neurons, *N* = number of independent experiments. ***H***, Representative composite confocal images of cortical neurons depicting NPY-pHluorin and Homer1 at DIV9 after 48 h of TTX treatment. Scale bars, 10 μm. ***I***, Quantified fluorescence intensity of DCV markers for every treated condition. NPY-pHluorin *p* = 5.87× 10^−06^, Homer *p* = 0.0179. The data are presented as the mean ± SEM. Mann–Whitney test, *p < 0.05, **p < 0.01, ***p < 0.001, ns *p* > 0.05.

Our data suggest that potentiation of DCV exocytosis upon TTX-induced network silencing is partially due to the accumulation of DCVs in neurites. We characterized the temporal profile by treating neurons with TTX for 4, 24, and 48 h before fixation at DIV21 (Fig. 3*A*) and analyzing DCV distribution by immunofluorescence for different DCV cargo comparing presumptive excitatory neurons (Fig. 3*B*, gray) and inhibitory neurons (Fig. 3*C*, red) with dendritic marker MAP-2 in blue (Fig. 3*B*,*C*). The majority of neurons in primary cortical cultures are excitatory (Delgado et al., 2022). In addition, we analyzed the subpopulation of GABAergic neurons expressing NPY (Fig. 3*C–E*). These neurons did not show an increase in NPY intensity upon network silencing after 4 h but exhibited a further increase after 24 h (3.3-fold) and 48 h (3.5-fold) (Fig. 3*E*,*F*, red) after normalization to control levels. We costained NPY-positive neurons for transmembrane marker IA-2 and observed a similar increase in IA-2 signal in neurites after 24 h (1.7-fold) and 48 h (1.6-fold), with no significant increase after only 4 h of silencing (Fig. 3*F*, orange). Next, we expressed NPY-pHluorin and colabeled presumptive excitatory neurons with ChgB or IA-2 (Figs. 2*A*, 3*B*). We observed a significant increase in ChgB and IA-2 intensities in presumptive excitatory neurons, after 4 h (ChgB 1.9-fold and IA-2 1.5-fold), but also at 24–48 h of silencing (Fig. 3*B*,*G*). The increase in NPY expression in GABAergic neurons (Fig. 3*F*) was significantly slower than the expression of DCV markers in the presumptive excitatory neuronal population (Fig. 3*G*). We confirmed this difference in timing between GABAergic and presumptive glutamatergic neurons by costaining NPY-positive neurons with the same DCV marker used in the presumptive excitatory population, IA-2. In inhibitory neurons, the highest peak was observed after 24 h (1.7-fold; Fig. 3*F*), whereas in mixed populations IA-2 exhibited a significant increase after only 4 h (1.5-fold) of TTX-induced network silencing (Fig. 3*G*). Thus, even short-term TTX-induced network silencing (4 h) increased the intensity of DCVs in the neurites of cortical neurons. This increase in DCV accumulation in neurites was also observed in different developmental stages of mouse cortical neurons, at DIV9, DIV15, and DIV21 (Figs. 2*A–C*, 3*F–I*). When fixing cells at DIV9, we colabeled NPY-pHluorin–positive cells with the postsynaptic marker Homer (Fig. 3*H*) known to be upregulated upon TTX-mediated silencing (Ehlers 2003). Forty-eight hours after TTX treatment, both, NPY-pHluorin and Homer, levels were increased (Fig. 3*I*).

### TTX-induced network silencing decreases transcription of neuropeptide genes

**Figure 4.**
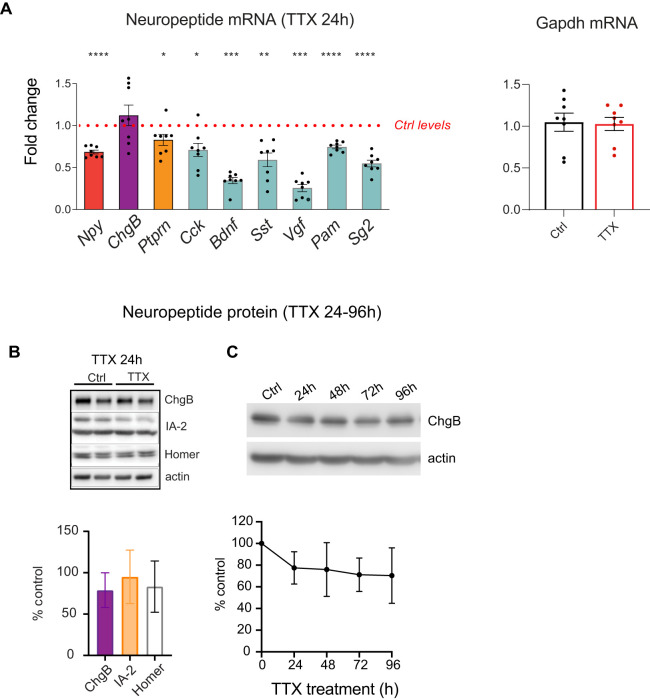
TTX-induced network silencing results in the decreased transcription of neuropeptide genes in mouse cortical neurons. ***A***, mRNA levels of different neuropeptide and DCV cargo transcripts of Ctrl and TTX-treated cortical neurons as assessed by quantitative RT-PCR and normalized to GAPDH. Data are shown as fold changes (FC) relative to control levels in the corresponding culture preparation. *n* = 8, *N* = 4 biological independent experiments. Dots represent individual cultures, bar graphs are geometric means, and error bars are geometric SD. Log2FC (ΔΔCt) were analyzed by one-sample *t* test. **p < 0.01; ***p < 0.001; ****p < 0.0001. ns, not significant. *n*, number of samples (wells); *N*, number of independent experiments. NPY *p* < 0.0001, ChgB *p* = 0.55, Ptprn *p* = 0.0326, CCK *p* = 0.0117, Bdnf *p* = 0.001, Sst *p* = 0.0091, Vgf *p* = 0.0001, Pam *p* < 0.0001, Scg2 *p* < 0.0001. ***B***, Quantification of ChgB, IA-2, and Homer1 levels as detected by Western blot (WB) in lysates of Ctrl and TTX-treated wild-type neurons for 24 h TTX treatment. Error bars represent SD. Data were analyzed using a two-tailed paired *t* test *n* = 3 biological independent experiments. ***C***, Chromogranin B (ChgB) and actin levels as detected by western blot (WB) in lysates of Ctrl and TTX-treated cortical neurons for four different timepoints of TTX treatment (1–4 d). Quantification of ChgB bands intensity from WB exemplified in ***C*** for four different timepoints of TTX treatment. Error bars represent SD. Data were analyzed using a two-tailed paired *t* test (4 biological independent experiments).

We next tested if the increased intensity of DCV cargos in response to network silencing depends on new mRNA synthesis. Thus, we investigated mRNA expression levels of the same DCV marker as tested before (NPY, ChgB, IA-2/Ptprn) with an additionally panel of common neuropeptides and general DCV components in cortical cultures (DIV14) silenced for 24 h. Unexpectedly, the mRNA levels of all neuropeptides and many general DCV markers were decreased after TTX treatment (Fig. 4*A*). *Vgf* and *Bdnf* showed the strongest reduction (∼65%), followed by *Sst and Scg2* (∼50%). mRNA levels of genes encoding cholecystokinin (*Cck*), *NPY*, and peptidylglycine alpha-amidating monooxygenase, processing enzyme (*Pam*), displayed a reduction of ∼30%. The decrease in mRNA was also pronounced for *Ptprn/IA-2* (∼20%), whereas *ChgB* mRNA levels were not significantly adjusted. Of note, *Gapdh* expression levels, used as a control, was not affected by TTX treatment (Fig. 4*A*). The general decrease in mRNA levels, in contrast to the increased protein signals detected by ICC, indicate that the TTX-induced accumulation of DCV cargos is not explained by transcriptional upregulation of the corresponding genes but that mRNA changes are in fact opposite to the changes observed at the protein level.

Finally, we asked whether the increased staining intensities of DCV cargos in the neurites of silenced neurons correlated with an overall increase in the total protein levels of these cargos, using Western blot analysis of lysed cultures (Fig. 4*B*,*C*). No increase in total protein levels of DCV cargo ChgB and Ptprn/IA-2 or postsynaptic protein Homer was observed in neurons treated with TTX for 24 h at DIV13 (Fig. 4*B*). Even prolonged silencing for 4 d (start TTX at DIV13) did not increase ChgB levels (Fig. 4*C*). These data suggest that TTX-induced increase in DCV cargos in neurites is not due to an overall increase in total cargo levels, but rather to an increased accumulation in DCVs.

### Neuronal network silencing by expression of TeNT also induces DCV accumulation in neurites

**Figure 5.**
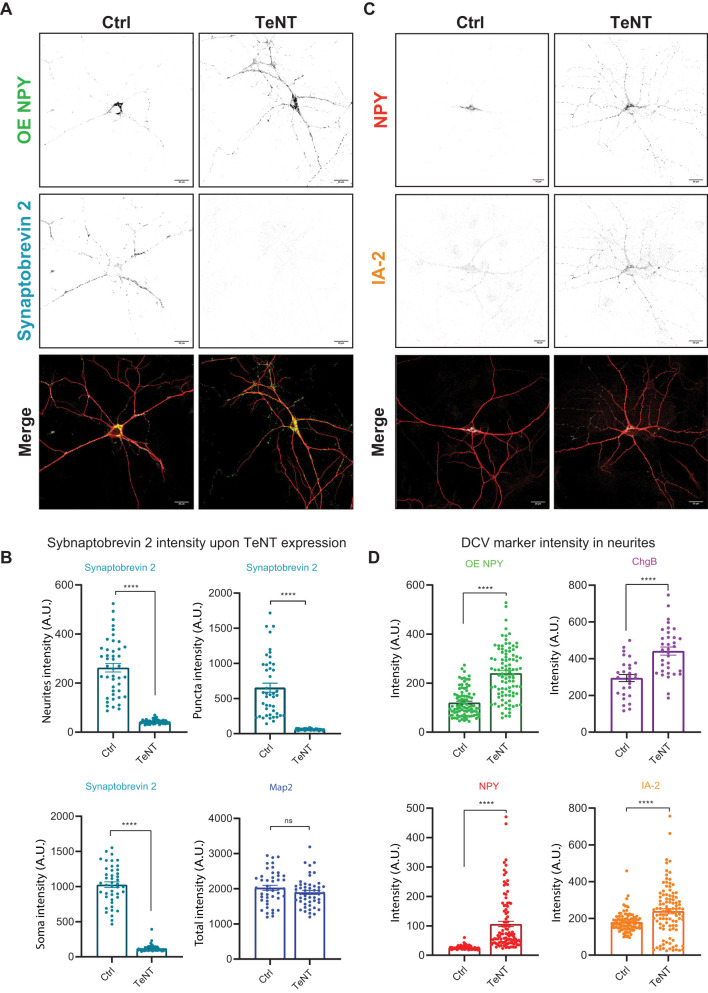
TeNT induced block of secretory vesicle exocytosis increases DCV marker intensity in mouse cortical neurons. ***A***, Representative composite confocal image of TeNT infected or control cortical neurons. Neurons were stained for Synaptobrevin2, MAP-2, and NPY-pHluorin; Scale bars, 10 μm. ***B***, Quantified fluorescence of Synaptobrevin2 in neurites, synapses, and soma of control and TeNT-expressing neurons. MAP-2 intensity is similar between Ctrl and TeNT infected neurons. The data are presented as the mean ± SEM. *p < 0.05; **p < 0.01; ***p < 0.001; and ****p < 0.0001. Synaptobrevin2 (*n* = 51. *N* = 3) control and TTX-treated neurons, *p* < 0.0001. MAP-2 *p* = 0.1788. ***C***, Representative composite confocal image of TeNT infected or control cortical neurons. Neurons were stained for NPY endogenous and IA-2. Scale bars, 10 μm. ***D***, Quantified fluorescence intensity of DCV markers in neurites of TeNT infected or control cortical neurons. The data are presented as the mean ± SEM. NPY-pHluorin (*n* = 96, *N* = 4) *p* = 1.73× 10^−15^, ChgB (*n* = 35, *N* = 3) *p* = 2.42× 10^−05^, NPY endogenous (*n* = 100, *N* = 4), Ia-2 (*n* = 100, *N* = 3) *p* = 3.15× 10^−05^, control and TTX-treated neurons. Mann–Whitney test, *p < 0.05, **p < 0.01, ***p < 0.001, ns *p* > 0.05. Dots represent individual neurons; *n*, number of neurons; *N*, number of independent experiments.

The observed accumulation of DCVs in neurites may be due to reduced exocytosis during a period of chronic network silencing. To test this possibility, we used an independent approach to inhibit exocytosis of both SV and DCV by overexpression of tetanus toxin (TeNT) light chain, which cleaves vesicular SNAREs Synaptobtrevin1, 2 and 3, necessary for the evoked exocytosis both types of neuronal vesicles therefore blocking synaptic activity (Montecucco and Schiavo 1994; Sakaba et al., 2005; Shimojo et al., 2015; Hoogstraaten et al., 2020). TeNT blocks synaptic vesicle exocytosis but allows action potentials to propagate through the neuron. TTX blocks all Na^+^ channel-dependent neuronal activity, preventing AP-induced exocytosis of both SVs and DCVs, though it does not affect spontaneous release. To test if TeNT expression phenocopies the observed DCV accumulation after TTX application, we expressed TeNT at DIV12, allowing a 2 d period of genomic integration and TeNT expression, and fixed at DIV15 to approximate the silencing period used in the TTX experiments. TeNT expression in cortical neurons resulted in a loss of synaptobrevin-2 signal as detected by ICC (Fig. 5*A*,*B*), and NPY-pHluorin intensity in dendrites of these neurons was increased by twofold in neurites (Fig. 5*A*,*B*). The levels of endogenously expressed NPY (∼4-fold), ChgB (1.5-fold), and IA-2 (1.3-fold) were increased in neurites of TeNT-expressing neurons (Fig. 5*C*,*D*). These data indicate that the accumulation of DCV cargos in neurites may be largely explained by reduced exocytosis during chronic network silencing.

## Discussion

Neuronal network activity is stabilized by regulating neuronal excitability and synaptic strength, for instance, by regulating synaptic vesicle release efficacy (Davis and Goodman 1998; Turrigiano and Nelson 2000; Marder and Prinz 2002; Perez-Otano and Ehlers 2005; Tien and Kerschensteiner 2018). Whether the release of neuropeptides and neurotrophic factors from DCVs is regulated in a similar fashion remains poorly understood. In this study, we examined mechanisms that regulate DCV exocytosis efficacy after TTX-mediated network silencing and found that silencing results in an increase in the total DCV pool, the accumulation of DCVs in neurites (Figs. 1–3) and a massive (700%) increase in the number of fusion events for a given stimulation pattern (Fig. 1) after network reactivation. The observed increase in fusion events was not fully explained by an increase in the total vesicle pool: the release fraction, the number of fusion events normalized to the remaining pool, was also increased in TTX-treated neurons (Fig. 1*E*). The accumulation of DCV cargos in neurites and the increase in DCV pool size were not a consequence of increased transcription/translation since total mRNA and proteins levels of these cargos were either unchanged or even decreased (Fig. 4).

Enhancing exocytosis efficacy in response to network silencing is a well-described and replicated aspect of homeostatic regulation for synaptic vesicle exocytosis in many synapses (Davis and Goodman 1998; Davis 2006; Moulder et al., 2006; Muller et al., 2012). The current study shows that the efficacy of the alternative regulated secretion pathway, which involves the secretion of neuropeptides and neurotrophins from DCVs, is also significantly enhanced (Fig. 1). Furthermore, the effect size for DCV exocytosis is remarkably larger, reaching 700% (Fig. 1), compared with the typical increase of 10–200% seen with SV exocytosis (Frank et al., 2006; Ortega et al., 2018). Hence, the massive potentiation of DCV exocytosis is a relatively large factor and may be explained by the fact that efficacy of DCV exocytosis is relatively low under resting conditions.

A number of presynaptic factors controlling homeostatic regulation of SV exocytosis efficacy have been identified, including a concerted action of proteins, BRP, RBP, RIM, (m)unc13, (m)unc18, syntaxin, and Fife (Jiang et al., 2010; Muller et al., 2012; Ortega et al., 2018; Toonen and Verhage 2022). Since many of these proteins are also important for DCV exocytosis (Arora et al., 2017; Yin et al., 2018; Persoon et al., 2019; Puntman et al., 2021), it seems plausible that these proteins are also involved in the homeostatic regulation of DCV exocytosis upon network silencing.

Increases in presynaptic calcium influx, upregulated Ca^2+^ currents, and an increase in the pool of release-ready synaptic vesicles (RRP) are central aspects in homeostatic regulation of synaptic vesicle release (Muller et al., 2012, 2015; Ratkai et al., 2021). The causal role of presynaptic calcium influx was demonstrated at the Drosophila NMJ, where a point mutation in the pore-forming α1 subunit of the CaV2.1 channel arrested homeostatic modulation of both calcium influx and neurotransmitter release (Smith et al., 1996, 1998; Frank et al., 2006; Muller and Davis 2012). DCV exocytosis also relies completely on calcium influx, and calcium influx was indeed also increased in response to silencing also in our experiments (Fig. 1*F–H*). The enhanced calcium responses after network silencing and reactivation may explain the enhanced exocytosis of DCVs, although we did not directly assess calcium dynamics in all the relevant locations in the neuron. Hence, the observed increase in DCV exocytosis efficacy may be explained, together with the increased efficacy of the release machinery shared with SV exocytosis, by homeostatic upregulation of calcium influx.

The observed increase in fusion efficacy may also be supported by increased pre-docking of DCV's prior to stimulation. A previous study observed more DCVs docked at active zones of rat neurons upon chronic network silencing (Tao et al., 2018), similar to the increase in the number of docked SVs and active zone area observed in hippocampal neurons (Chipman et al., 2022). At the *Drosophila* neuromuscular junction, activity stimulates synaptic capture of DCVs, increases retrograde transport, and enhances mobility of DCVs for faster transmission (Shakiryanova et al., 2006; Levitan 2008; Cavolo et al., 2016). The kinetics and efficacy of the DCV docking process are unknown but may be a rate-limiting factor. During network silencing, vesicles may have more time to dock and set up all molecular interactions required for exocytosis, resulting in more fusogenic DCVs after a period of activity suppression.

The enhanced DCV exocytosis efficacy after network silencing was accompanied by an increased staining intensity for endogenous DCV markers (Figs. 2, 3), suggesting an increased DCV production that probably contributes to enhanced DCV exocytosis efficacy after network silencing. Homeostatic synaptic plasticity is known to involve transcriptional regulation and new protein synthesis, usually by the production of new mRNAs which are transported throughout the neuron (Ibata et al., 2008; Han and Stevens 2009; Schaukowitch et al., 2017). However, we did not observe transcriptional upregulation of DCV marker genes and, except ChgB, DCV marker transcripts were in fact lower upon network silencing, despite higher staining intensities for the corresponding proteins in neurites (Fig. 4*A*) and mostly unchanged total protein levels in WB (Fig. 4*B*,*C*).

While mRNA levels of *Ptprn* were decreased upon silencing of neuronal activity, total levels of the encoded protein IA-2 were unchanged in Western blot analysis. This can be explained by the fact that downregulation of transcription is compensated by a decreased IA-2 secretion during TTX. For other peptides, it was not possible to obtain independent confirmation of the immunocytochemistry data since the quantification of most processed neuropeptides such as NPY is challenging by WB analysis due to their small size and low abundancy (Subkhangulova et al., 2023). The higher staining intensities may be explained by increased local translation of existing mRNAs (Steward and Schuman 2003; Martin and Zukin 2006; Bramham and Wells 2007), redistribution of DCV marker proteins into neurites, and/or a prolonged half-life of proteins (reduced local protein degradation (Dorrbaum et al., 2020). In addition, when using TeNT to inhibit both SV and DCV exocytosis, we observed similar accumulation of DCV markers in neurites as with neuronal silencing with TTX (Fig. 5). The effect size was even slightly higher than after TTX-induced silencing, possibly due to longer and also complete network silencing, thereby inhibiting the vesicle fusion reaction. Hence, the increased accumulation of DCVs in neurites may be explained by reduced exocytosis of DCVs due to silencing, rather than homeostatic upregulation of DCV cargo.

The significant reduction in mRNA levels for neuropeptide genes after network silencing (Fig. 4) suggests activity-dependent regulation of neuropeptide gene expression. Indeed, in the opposite situation, increasing network activity induced the expression of most neuropeptides (Schaukowitch et al., 2017). Cleaved neuropeptide domains probably form a signaling feedback loop to the nucleus regulating transcription (Subramaniam et al., 1993; Trajkovski et al., 2004; Vo et al., 2004; Ferraro et al., 2005; Francone et al., 2010). For instance, in pancreatic beta-cells, exocytosis of secretory granules leads to insulin release but also to insertion of the transmembrane DCV protein Ptprn/ICA512 in the plasma membrane and the [Ca^2+^]-dependent cleavage of ICA512 cytoplasmic domain by mu-calpain. The ICA512 cytosolic fragment is targeted to the nucleus and upregulates insulin expression (Trajkovski et al., 2004). Such feedback signaling is expected to be largely absent during network silencing, as in the absence of activity, DCV exocytosis is very low (Laurent et al., 2018; Moro et al., 2021b). Additionally, overexpression of the ICA512 cytosolic fragment increased the mRNA expression levels of neuropeptide genes, ICA512 and insulin itself (Trajkovski et al., 2004; Mziaut et al., 2006). This suggests a homeostatic downregulation of DCV cargo due to lack of fusion and retrograde signaling to the nucleus, additionally resulting in DCV accumulation in neurites. One study of the essential secretory granule membrane enzyme PAM revealed a pathway that relays information from secretory granules to the nucleus, resulting in alterations in gene expression (Francone et al., 2010).

Additionally, low calcium levels during network silencing could also contribute to the decreased mRNA levels, as expression of at least some DCV cargo is Ca^2+^ dependent, for instance, BDNF (Castren et al., 1998; Fujita et al., 1999; Tao et al., 2002; Lin et al., 2015). BDNF also enhances expression of various neuropeptides in cultured neocortical neurons. BDNF and neurotrophin-5 (NT-5) increased the levels of NPY and Sst, while neurotrophin-3 (NT-3) also increased these peptides but required higher concentrations (Carnahan and Nawa 1995). Also, the 3′UTR of Vgf mRNA regulates its own translational efficiency and Vgf has a role in regulating granin levels (Lin et al., 2021).

Chronic network silencing with TTX elicits distinct responses in glutamatergic and GABAergic synapses to maintain neuronal homeostasis (Kilman et al., 2002; Hartmann et al., 2008; Gronborg et al., 2010; Kim et al., 2024). At glutamatergic synapses, TTX enhances presynaptic release probability and spontaneous release events, often through increased synaptic vesicle recruitment and priming, and hereby sustains excitatory drive (Lee et al., 2008, 2012; Korber and Kuner 2016). Conversely, GABAergic synapses typically exhibit reduced vesicle release probability and spontaneous release frequency, partly due to differences in vesicle mobilization and synaptic protein interactions, such as those involving vesicular glutamate (VGLUT) and GABA transporters (VGAT; Kilman et al., 2002; Hartmann et al., 2008; Gronborg et al., 2010; Kim et al., 2024). Whether differences between glutamatergic and GABAergic neurons also exist for the TTX-dependent regulation of DCV exocytosis needs further investigation. Our DCV exocytosis reporter, NPY-pHluorin, was expressed ubiquitously in mixed cultures containing both glutamatergic and GABAergic neurons (Persoon et al., 2018).

Current understanding of synaptic homeostatic mechanisms suggests that they help maintain network stability by restoring synaptic strength to a predetermined set point following activity changes (Davis and Goodman 1998; Turrigiano 1999; Davis and Bezprozvanny 2001; Murthy et al., 2001). However, it is not immediately clear how the significant increase in DCV exocytosis due to accumulation after silencing observed in this study contributes to this process. A chronic increase in the activity of PV+ interneurons leads to the cell-autonomous transcriptional upregulation of two neuropeptides, Vgf and Scg2, the former being critically required for the activity-dependent scaling of inhibitory synapses (Selten et al., 2023). On the other hand, neuropeptides elicit a plethora of effects in receiving neurons, both enhancing and inhibiting activity and on different time scales. Our detection method using the heterologous reporter NPY-pHluorin ensures that our assay detects the release of all neuropeptides and neurotrophins accumulated in DCVs, because the reporter accumulates together with endogenous peptides/neurotrophins in >80% of all DCVs in neurons studied so far without changing DCV biogenesis (Farina et al., 2015; van Keimpema et al., 2017; Dominguez et al., 2018; Persoon et al., 2018; Moro et al., 2021b). Hence, it is unlikely that the observed potentiation of DCV exocytosis leads to the selective secretion of specific signals, for instance activity-enhancing signals. Instead, the massive potentiation of neuropeptide release from DCVs, upon reactivation after network silencing might contribute to a new dimension of metaplasticity, enhancing a variety of modulatory pathways in neuronal networks rather than selectively bringing synaptic strength back to a prior set point.

## Synthesis

Reviewing Editor: Eran Perlson, Tel Aviv University Decisions are customarily a result of the Reviewing Editor and the peer reviewers coming together and discussing their recommendations until a consensus is reached. When revisions are invited, a fact-based synthesis statement explaining their decision and outlining what is needed to prepare a revision will be listed below. The following reviewer(s) agreed to reveal their identity: NONE. Note: If this manuscript was transferred from JNeurosci and a decision was made to accept the manuscript without peer review, a brief statement to this effect will instead be what is listed below.

I have read the manuscript and the reviewers' comments. Both reviewers agree that the manuscript has been significantly improved and that many initial concerns have been addressed. However, there are still outstanding issues that need to be resolved. I believe the manuscript can be accepted for publication after these comments are addressed, even if this involves adding to the discussion sections a paragraph on the limitation and future work addressing these issues, making authors conclusions less conclusive.

Key points to address even by modifying the text:

1. Novelty and Homeostasis. Address the concern that the observed increase in vesicle release is due to accumulation rather than an active process of upregulation. Clarify the interpretation in the discussion. Acknowledge that the study does not directly demonstrate homeostatic mechanisms and discuss this limitation. Consider expanding on how downstream signaling cascades or protein maintenance might be investigated in future studies to validate homeostasis.

2. Technical and Data Accuracy. Correct inconsistencies between the text and graph values in Fig 3F/G (lines 259, 262-3, and 268-9). Revise Fig 5B to improve clarity, for example, by labeling synaptobrevin consistently across all graphs.

3. Experimental Issues.

-Provide an explanation or correction for lines 230-237 regarding network activity in the presence of TTX

-Clarify in line 238 that the study is measuring DCV cargo labeling, not strict accumulation

-Acknowledge in the discussion that TTX does not universally increase vesicle fusion, as GABA SV release decreases with TTX.

4. Validation of mRNA Changes. Address the gap in Fig 4 by explaining why protein validation for mRNA changes was not conducted or cannot be performed. Expand the discussion to acknowledge this limitation.

## References

[B1] Arora S, Saarloos I, Kooistra R, van de Bospoort R, Verhage M, Toonen RF (2017) SNAP-25 gene family members differentially support secretory vesicle fusion. J Cell Sci 130:1877–1889. 10.1242/jcs.201889 28404788

[B2] Baginska U, Moro A, Toonen RF, Verhage M (2023) Maximal fusion capacity and efficient replenishment of the dense core vesicle pool in hippocampal neurons. J Neurosci 43:7616–7625. 10.1523/JNEUROSCI.2251-22.2023 37852790 PMC10634579

[B3] Bouwman J, Spijker S, Schut D, Wachtler B, Ylstra B, Smit AB, Verhage M (2006) Reduced expression of neuropeptide genes in a genome-wide screen of a secretion-deficient mouse. J Neurochem 99:84–96. 10.1111/j.1471-4159.2006.04041.x 16987237

[B4] Bramham CR, Wells DG (2007) Dendritic mRNA: transport, translation and function. Nat Rev Neurosci 8:776–789. 10.1038/nrn2150 17848965

[B5] Branco T, Staras K, Darcy KJ, Goda Y (2008) Local dendritic activity sets release probability at hippocampal synapses. Neuron 59:475–485. 10.1016/j.neuron.2008.07.006 18701072 PMC6390949

[B6] Burrone J, Murthy VN (2003) Synaptic gain control and homeostasis. Curr Opin Neurobiol 13:560–567. 10.1016/j.conb.2003.09.007 14630218

[B7] Carnahan J, Nawa H (1995) Regulation of neuropeptide expression in the brain by neurotrophins: potential role in vivo. Mol Neurobiol 10:135–149. 10.1007/BF02740672 7576304

[B8] Castren E, Berninger B, Leingartner A, Lindholm D (1998) Regulation of brain-derived neurotrophic factor mRNA levels in hippocampus by neuronal activity. Prog Brain Res 117:57–64. 10.1016/S0079-6123(08)64007-8 9932400

[B9] Cavolo SL, Bulgari D, Deitcher DL, Levitan ES (2016) Activity induces Fmr1-sensitive synaptic capture of anterograde circulating neuropeptide vesicles. J Neurosci 36:11781–11787. 10.1523/JNEUROSCI.2212-16.2016 27852784 PMC5125230

[B10] Chemelli RM, et al. (1999) Narcolepsy in orexin knockout mice: molecular genetics of sleep regulation. Cell 98:437–451. 10.1016/S0092-8674(00)81973-X 10481909

[B11] Chen R, Chung SH (2014) Mechanism of tetrodotoxin block and resistance in sodium channels. Biochem Biophys Res Commun 446:370–374. 10.1016/j.bbrc.2014.02.115 24607901

[B12] Chipman PH, Fetter RD, Panzera LC, Bergerson SJ, Karmelic D, Yokoyama S, Hoppa MB, Davis GW (2022) NMDAR-dependent presynaptic homeostasis in adult hippocampus: synapse growth and cross-modal inhibitory plasticity. Neuron 110:3302–3317.e7. 10.1016/j.neuron.2022.08.014 36070750 PMC9588671

[B13] Davis GW (2006) Homeostatic control of neural activity: from phenomenology to molecular design. Annu Rev Neurosci 29:307–323. 10.1146/annurev.neuro.28.061604.135751 16776588

[B14] Davis GW, Bezprozvanny I (2001) Maintaining the stability of neural function: a homeostatic hypothesis. Annu Rev Physiol 63:847–869. 10.1146/annurev.physiol.63.1.847 11181978

[B15] Davis GW, Goodman CS (1998) Genetic analysis of synaptic development and plasticity: homeostatic regulation of synaptic efficacy. Curr Opin Neurobiol 8:149–156. 10.1016/S0959-4388(98)80018-4 9568402

[B16] Delgado RN, Allen DE, Keefe MG, Mancia Leon WR, Ziffra RS, Crouch EE, Alvarez-Buylla A, Nowakowski TJ (2022) Individual human cortical progenitors can produce excitatory and inhibitory neurons. Nature 601:397–403. 10.1038/s41586-021-04230-7 34912114 PMC8994470

[B17] de Wit J, Toonen RF, Verhage M (2009) Matrix-dependent local retention of secretory vesicle cargo in cortical neurons. J Neurosci 29:23–37. 10.1523/JNEUROSCI.3931-08.2009 19129381 PMC6664920

[B18] Dominguez N, van Weering JRT, Borges R, Toonen RFG, Verhage M (2018) Dense-core vesicle biogenesis and exocytosis in neurons lacking chromogranins A and B. J Neurochem 144:241–254. 10.1111/jnc.14263 29178418 PMC5814729

[B19] Dorrbaum AR, Alvarez-Castelao B, Nassim-Assir B, Langer JD, Schuman EM (2020) Proteome dynamics during homeostatic scaling in cultured neurons. Elife 9:e52939. 10.7554/eLife.52939 32238265 PMC7117909

[B20] Ehlers MD (2003) Activity level controls postsynaptic composition and signaling via the ubiquitin-proteasome system. Nat Neurosci 6:231–242. 10.1038/nn1013 12577062

[B21] Farina M, van de Bospoort R, He E, Persoon CM, van Weering JR, Broeke JH, Verhage M, Toonen RF (2015) CAPS-1 promotes fusion competence of stationary dense-core vesicles in presynaptic terminals of mammalian neurons. Elife 4:e05438. 10.7554/elife.05438 25719439 PMC4341531

[B22] Ferraro F, Eipper BA, Mains RE (2005) Retrieval and reuse of pituitary secretory granule proteins. J Biol Chem 280:25424–25435. 10.1074/jbc.M414156200 15905171

[B23] Francone VP, Ifrim MF, Rajagopal C, Leddy CJ, Wang Y, Carson JH, Mains RE, Eipper BA (2010) Signaling from the secretory granule to the nucleus: uhmk1 and PAM. Mol Endocrinol 24:1543–1558. 10.1210/me.2009-0381 20573687 PMC2940467

[B24] Frank CA, Kennedy MJ, Goold CP, Marek KW, Davis GW (2006) Mechanisms underlying the rapid induction and sustained expression of synaptic homeostasis. Neuron 52:663–677. 10.1016/j.neuron.2006.09.029 17114050 PMC2673733

[B25] Fujita Y, Katagi J, Tabuchi A, Tsuchiya T, Tsuda M (1999) Coactivation of secretogranin-II and BDNF genes mediated by calcium signals in mouse cerebellar granule cells. Brain Res Mol Brain Res 63:316–324. 10.1016/S0169-328X(98)00299-X 9878806

[B26] Gronborg M, Pavlos NJ, Brunk I, Chua JJ, Munster-Wandowski A, Riedel D, Ahnert-Hilger G, Urlaub H, Jahn R (2010) Quantitative comparison of glutamatergic and GABAergic synaptic vesicles unveils selectivity for few proteins including MAL2, a novel synaptic vesicle protein. J Neurosci 30:2–12. 10.1523/JNEUROSCI.4074-09.2010 20053882 PMC6632534

[B27] Han EB, Stevens CF (2009) Development regulates a switch between post- and presynaptic strengthening in response to activity deprivation. Proc Natl Acad Sci U S A 106:10817–10822. 10.1073/pnas.0903603106 19509338 PMC2705571

[B28] Hartmann K, Bruehl C, Golovko T, Draguhn A (2008) Fast homeostatic plasticity of inhibition via activity-dependent vesicular filling. PLoS One 3:e2979. 10.1371/journal.pone.0002979 18714334 PMC2495031

[B29] Hoogstraaten RI, van Keimpema L, Toonen RF, Verhage M (2020) Tetanus insensitive VAMP2 differentially restores synaptic and dense core vesicle fusion in tetanus neurotoxin treated neurons. Sci Rep 10:10913. 10.1038/s41598-020-67988-2 32616842 PMC7331729

[B30] Ibata K, Sun Q, Turrigiano GG (2008) Rapid synaptic scaling induced by changes in postsynaptic firing. Neuron 57:819–826. 10.1016/j.neuron.2008.02.031 18367083

[B31] Jiang X, Litkowski PE, Taylor AA, Lin Y, Snider BJ, Moulder KL (2010) A role for the ubiquitin-proteasome system in activity-dependent presynaptic silencing. J Neurosci 30:1798–1809. 10.1523/JNEUROSCI.4965-09.2010 20130189 PMC2824895

[B32] Kilman V, van Rossum MC, Turrigiano GG (2002) Activity deprivation reduces miniature IPSC amplitude by decreasing the number of postsynaptic GABA(A) receptors clustered at neocortical synapses. J Neurosci 22:1328–1337. 10.1523/JNEUROSCI.22-04-01328.2002 11850460 PMC6757564

[B33] Kim JH, et al. (2024) GABAergic/glycinergic and glutamatergic neurons mediate distinct neurodevelopmental phenotypes of STXBP1 encephalopathy. J Neurosci 44:1806–1823. 10.1523/JNEUROSCI.1806-23.2024 38360746 PMC10993039

[B34] Korber C, Kuner T (2016) Molecular machines regulating the release probability of synaptic vesicles at the active zone. Front Synaptic Neurosci 8:5. 10.3389/fnsyn.2016.00005 26973506 PMC4773589

[B35] Laurent P, Ch'ng Q, Jospin M, Chen C, Lorenzo R, de Bono M (2018) Genetic dissection of neuropeptide cell biology at high and low activity in a defined sensory neuron. Proc Natl Acad Sci U S A 115:E6890–E99. 10.1073/pnas.1714610115 29959203 PMC6055185

[B37] Lee JS, Kim MH, Ho WK, Lee SH (2008) Presynaptic release probability and readily releasable pool size are regulated by two independent mechanisms during posttetanic potentiation at the calyx of Held synapse. J Neurosci 28:7945–7953. 10.1523/JNEUROSCI.2165-08.2008 18685020 PMC6670772

[B36] Lee JS, Ho WK, Lee SH (2012) Actin-dependent rapid recruitment of reluctant synaptic vesicles into a fast-releasing vesicle pool. Proc Natl Acad Sci U S A 109:E765–74. 10.1073/pnas.1114072109 22393020 PMC3323990

[B38] Levitan ES (2008) Signaling for vesicle mobilization and synaptic plasticity. Mol Neurobiol 37:39–43. 10.1007/s12035-008-8014-3 18446451 PMC2398727

[B39] Lin WJ, et al. (2021) An increase in VGF expression through a rapid, transcription-independent, autofeedback mechanism improves cognitive function. Transl Psychiatry 11:383. 10.1038/s41398-021-01489-2 34238925 PMC8266826

[B40] Lin WJ, Jiang C, Sadahiro M, Bozdagi O, Vulchanova L, Alberini CM, Salton SR (2015) VGF and Its C-terminal peptide TLQP-62 regulate memory formation in hippocampus via a BDNF-TrkB-dependent mechanism. J Neurosci 35:10343–10356. 10.1523/JNEUROSCI.0584-15.2015 26180209 PMC4502270

[B41] Lissin DV, Gomperts SN, Carroll RC, Christine CW, Kalman D, Kitamura M, Hardy S, Nicoll RA, Malenka RC, von Zastrow M (1998) Activity differentially regulates the surface expression of synaptic AMPA and NMDA glutamate receptors. Proc Natl Acad Sci U S A 95:7097–7102. 10.1073/pnas.95.12.7097 9618545 PMC22752

[B42] Liu Y, et al. (2023) Oxytocin promotes prefrontal population activity via the PVN-PFC pathway to regulate pain. Neuron 111:1795–1811.e7. 10.1016/j.neuron.2023.03.014 37023755 PMC10272109

[B43] MacArthur L, Eiden L (1996) Neuropeptide genes: targets of activity-dependent signal transduction. Peptides 17:721–728. 10.1016/0196-9781(95)02100-0 8804085

[B44] Marder E, Goaillard JM (2006) Variability, compensation and homeostasis in neuron and network function. Nat Rev Neurosci 7:563–574. 10.1038/nrn1949 16791145

[B45] Marder E, Prinz AA (2002) Modeling stability in neuron and network function: the role of activity in homeostasis. Bioessays 24:1145–1154. 10.1002/bies.10185 12447979

[B46] Martin KC, Zukin RS (2006) RNA trafficking and local protein synthesis in dendrites: an overview. J Neurosci 26:7131–7134. 10.1523/JNEUROSCI.1801-06.2006 16822966 PMC6673931

[B47] Mennerick S, Que J, Benz A, Zorumski CF (1995) Passive and synaptic properties of hippocampal neurons grown in microcultures and in mass cultures. J Neurophysiol 73:320–332. 10.1152/jn.1995.73.1.320 7714575

[B48] Montecucco C, Schiavo G (1994) Mechanism of action of tetanus and botulinum neurotoxins. Mol Microbiol 13:1–8. 10.1111/j.1365-2958.1994.tb00396.x 7527117

[B49] Moro A, Hoogstraaten RI, Persoon CM, Verhage M, Toonen RF (2021a) Quantitative analysis of dense-core vesicle fusion in rodent CNS neurons. STAR Protoc 2:100325. 10.1016/j.xpro.2021.100325 33659902 PMC7890040

[B50] Moro A, van Nifterick A, Toonen RF, Verhage M (2021b) Dynamin controls neuropeptide secretion by organizing dense-core vesicle fusion sites. Sci Adv 7:eabf0659. 10.1126/sciadv.abf0659 34020952 PMC8139595

[B51] Moulder KL, Jiang X, Taylor AA, Olney JW, Mennerick S (2006) Physiological activity depresses synaptic function through an effect on vesicle priming. J Neurosci 26:6618–6626. 10.1523/JNEUROSCI.5498-05.2006 16775150 PMC6674037

[B52] Moulder KL, Meeks JP, Shute AA, Hamilton CK, de Erausquin G, Mennerick S (2004) Plastic elimination of functional glutamate release sites by depolarization. Neuron 42:423–435. 10.1016/S0896-6273(04)00184-9 15134639

[B53] Muller M, Davis GW (2012) Transsynaptic control of presynaptic Ca(2)(+) influx achieves homeostatic potentiation of neurotransmitter release. Curr Biol 22:1102–1108. 10.1016/j.cub.2012.04.018 22633807 PMC4367479

[B54] Muller M, Genc O, Davis GW (2015) RIM-binding protein links synaptic homeostasis to the stabilization and replenishment of high release probability vesicles. Neuron 85:1056–1069. 10.1016/j.neuron.2015.01.024 25704950 PMC4354699

[B55] Muller M, Liu KS, Sigrist SJ, Davis GW (2012) RIM controls homeostatic plasticity through modulation of the readily-releasable vesicle pool. J Neurosci 32:16574–16585. 10.1523/JNEUROSCI.0981-12.2012 23175813 PMC3523185

[B56] Murthy VN, Schikorski T, Stevens CF, Zhu Y (2001) Inactivity produces increases in neurotransmitter release and synapse size. Neuron 32:673–682. 10.1016/S0896-6273(01)00500-1 11719207

[B57] Mziaut H, et al. (2006) Synergy of glucose and growth hormone signalling in islet cells through ICA512 and STAT5. Nat Cell Biol 8:435–445. 10.1038/ncb1395 16622421

[B58] Nagasawa M, Mitsui S, En S, Ohtani N, Ohta M, Sakuma Y, Onaka T, Mogi K, Kikusui T (2015) Social evolution. Oxytocin-gaze positive loop and the coevolution of human-dog bonds. Science 348:333–336. 10.1126/science.1261022 25883356

[B59] Naldini L, Blomer U, Gallay P, Ory D, Mulligan R, Gage FH, Verma IM, Trono D (1996) In vivo gene delivery and stable transduction of nondividing cells by a lentiviral vector. Science 272:263–267. 10.1126/science.272.5259.263 8602510

[B60] Nurrish S (2014) Dense core vesicle release: controlling the where as well as the when. Genetics 196:601–604. 10.1534/genetics.113.159905 24653208 PMC3948793

[B61] O'Brien RJ, Kamboj S, Ehlers MD, Rosen KR, Fischbach GD, Huganir RL (1998) Activity-dependent modulation of synaptic AMPA receptor accumulation. Neuron 21:1067–1078. 10.1016/S0896-6273(00)80624-8 9856462

[B62] Orr BO, Fetter RD, Davis GW (2022) Activation and expansion of presynaptic signaling foci drives presynaptic homeostatic plasticity. Neuron 110:3743–3759.e6. 10.1016/j.neuron.2022.08.016 36087584 PMC9671843

[B63] Ortega JM, Genc O, Davis GW (2018) Molecular mechanisms that stabilize short term synaptic plasticity during presynaptic homeostatic plasticity. Elife 7:e40385. 10.7554/eLife.40385 30422113 PMC6250423

[B64] Perez-Otano I, Ehlers MD (2005) Homeostatic plasticity and NMDA receptor trafficking. Trends Neurosci 28:229–238. 10.1016/j.tins.2005.03.004 15866197

[B66] Persoon CM, Moro A, Nassal JP, Farina M, Broeke JH, Arora S, Dominguez N, van Weering JR, Toonen RF, Verhage M (2018) Pool size estimations for dense-core vesicles in mammalian CNS neurons. EMBO J 37:e99672. 10.15252/embj.201899672 30185408 PMC6187028

[B65] Persoon CM, Hoogstraaten RI, Nassal JP, van Weering JRT, Kaeser PS, Toonen RF, Verhage M (2019) The RAB3-RIM pathway is essential for the release of neuromodulators. Neuron 104:1065–1080.e12. 10.1016/j.neuron.2019.09.015 31679900 PMC6923582

[B67] Puntman DC, Arora S, Farina M, Toonen RF, Verhage M (2021) Munc18-1 is essential for neuropeptide secretion in neurons. J Neurosci 41:5980–5993. 10.1523/JNEUROSCI.3150-20.2021 34103363 PMC8276746

[B68] Rao A, Craig AM (1997) Activity regulates the synaptic localization of the NMDA receptor in hippocampal neurons. Neuron 19:801–812. 10.1016/S0896-6273(00)80962-9 9354327

[B69] Ratkai A, Tarnok K, Aouad HE, Micska B, Schlett K, Szucs A (2021) Homeostatic plasticity and burst activity are mediated by hyperpolarization-activated cation currents and T-type calcium channels in neuronal cultures. Sci Rep 11:3236. 10.1038/s41598-021-82775-3 33547341 PMC7864958

[B70] Sakaba T, Stein A, Jahn R, Neher E (2005) Distinct kinetic changes in neurotransmitter release after SNARE protein cleavage. Science 309:491–494. 10.1126/science.1112645 16020741

[B71] Schanzenbacher CT, Sambandan S, Langer JD, Schuman EM (2016) Nascent proteome remodeling following homeostatic scaling at hippocampal synapses. Neuron 92:358–371. 10.1016/j.neuron.2016.09.058 27764671 PMC5078608

[B72] Schaukowitch K, Reese AL, Kim SK, Kilaru G, Joo JY, Kavalali ET, Kim TK (2017) An intrinsic transcriptional program underlying synaptic scaling during activity suppression. Cell Rep 18:1512–1526. 10.1016/j.celrep.2017.01.033 28178527 PMC5524384

[B73] Selten M, Bernard C, Hamid F, Hanusz-Godoy A, Oozeer F, Zimmer C, Marín O (2023) Regulation of parvalbumin interneuron plasticity by neuropeptide-encoding genes. *bioRxiv*: 2023.02.03.527010. 10.1038/s41586-025-08933-z PMC12222018 40307547

[B74] Shakiryanova D, Tully A, Levitan ES (2006) Activity-dependent synaptic capture of transiting peptidergic vesicles. Nat Neurosci 9:896–900. 10.1038/nn1719 16767091

[B75] Shimojo M, Courchet J, Pieraut S, Torabi-Rander N, Sando R 3rd, Polleux F, Maximov A (2015) SNAREs controlling vesicular release of BDNF and development of callosal axons. Cell Rep 11:1054–1066. 10.1016/j.celrep.2015.04.032 25959820 PMC4439258

[B76] Smith LA, Peixoto AA, Kramer EM, Villella A, Hall JC (1998) Courtship and visual defects of cacophony mutants reveal functional complexity of a calcium-channel alpha1 subunit in *Drosophila*. Genetics 149:1407–1426. 10.1093/genetics/149.3.1407 9649530 PMC1460251

[B77] Smith LA, Wang X, Peixoto AA, Neumann EK, Hall LM, Hall JC (1996) A Drosophila calcium channel alpha1 subunit gene maps to a genetic locus associated with behavioral and visual defects. J Neurosci 16:7868–7879. 10.1523/JNEUROSCI.16-24-07868.1996 8987815 PMC6579206

[B78] Steward O, Schuman EM (2003) Compartmentalized synthesis and degradation of proteins in neurons. Neuron 40:347–359. 10.1016/S0896-6273(03)00635-4 14556713

[B79] Subkhangulova A, Gonzalez-Lozano MA, Groffen AJA, van Weering JRT, Smit AB, Toonen RF, Verhage M (2023) Tomosyn affects dense core vesicle composition but not exocytosis in mammalian neurons. Elife 12:e85561. 10.7554/eLife.85561 37695731 PMC10495110

[B80] Subramaniam M, Koedam JA, Wagner DD (1993) Divergent fates of P- and E-selectins after their expression on the plasma membrane. Mol Biol Cell 4:791–801. 10.1091/mbc.4.8.791 7694691 PMC300993

[B81] Tao CL, Liu YT, Zhou ZH, Lau PM, Bi GQ (2018) Accumulation of dense core vesicles in hippocampal synapses following chronic inactivity. Front Neuroanat 12:48. 10.3389/fnana.2018.00048 29942253 PMC6004418

[B82] Tao X, West AE, Chen WG, Corfas G, Greenberg ME (2002) A calcium-responsive transcription factor, CaRF, that regulates neuronal activity-dependent expression of BDNF. Neuron 33:383–395. 10.1016/S0896-6273(01)00561-X 11832226

[B83] Tien NW, Kerschensteiner D (2018) Homeostatic plasticity in neural development. Neural Dev 13:9. 10.1186/s13064-018-0105-x 29855353 PMC5984303

[B84] Toonen RF, Verhage M (2022) Homing in on homeostatic plasticity. Neuron 110:3645–3647. 10.1016/j.neuron.2022.10.033 36395749

[B85] Trajkovski M, Mziaut H, Altkruger A, Ouwendijk J, Knoch KP, Muller S, Solimena M (2004) Nuclear translocation of an ICA512 cytosolic fragment couples granule exocytosis and insulin expression in beta-cells. J Cell Biol 167:1063–1074. 10.1083/jcb.200408172 15596545 PMC2172607

[B86] Turrigiano GG (1999) Homeostatic plasticity in neuronal networks: the more things change, the more they stay the same. Trends Neurosci 22:221–227. 10.1016/S0166-2236(98)01341-1 10322495

[B87] Turrigiano GG (2008) The self-tuning neuron: synaptic scaling of excitatory synapses. Cell 135:422–435. 10.1016/j.cell.2008.10.008 18984155 PMC2834419

[B88] Turrigiano GG, Nelson SB (2000) Hebb and homeostasis in neuronal plasticity. Curr Opin Neurobiol 10:358–364. 10.1016/S0959-4388(00)00091-X 10851171

[B89] van Keimpema L, Kooistra R, Toonen RF, Verhage M (2017) CAPS-1 requires its C2, PH, MHD1 and DCV domains for dense core vesicle exocytosis in mammalian CNS neurons. Sci Rep 7:10817. 10.1038/s41598-017-10936-4 28883501 PMC5589909

[B90] Verhage M, McMahon HT, Ghijsen WE, Boomsma F, Scholten G, Wiegant VM, Nicholls DG (1991) Differential release of amino acids, neuropeptides, and catecholamines from isolated nerve terminals. Neuron 6:517–524. 10.1016/0896-6273(91)90054-4 2015091

[B91] Vo YP, Hutton JC, Angleson JK (2004) Recycling of the dense-core vesicle membrane protein phogrin in Min6 beta-cells. Biochem Biophys Res Commun 324:1004–1010. 10.1016/j.bbrc.2004.09.147 15485654

[B92] Wasmeier C, Hutton JC (1996) Molecular cloning of phogrin, a protein-tyrosine phosphatase homologue localized to insulin secretory granule membranes. J Biol Chem 271:18161–18170. 10.1074/jbc.271.30.18161 8663434

[B93] Wierda KD, Toonen RF, de Wit H, Brussaard AB, Verhage M (2007) Interdependence of PKC-dependent and PKC-independent pathways for presynaptic plasticity. Neuron 54:275–290. 10.1016/j.neuron.2007.04.001 17442248

[B94] Yin P, Gandasi NR, Arora S, Omar-Hmeadi M, Saras J, Barg S (2018) Syntaxin clusters at secretory granules in a munc18-bound conformation. Mol Biol Cell 29:2700–2708. 10.1091/mbc.E17-09-0541 30156474 PMC6249827

[B95] Yu LM, Goda Y (2009) Dendritic signalling and homeostatic adaptation. Curr Opin Neurobiol 19:327–335. 10.1016/j.conb.2009.07.002 19640698

[B96] Zachariou V, Brunzell DH, Hawes J, Stedman DR, Bartfai T, Steiner RA, Wynick D, Langel U, Picciotto MR (2003) The neuropeptide galanin modulates behavioral and neurochemical signs of opiate withdrawal. Proc Natl Acad Sci U S A 100:9028–9033. 10.1073/pnas.1533224100 12853567 PMC166432

[B97] Zhao C, Dreosti E, Lagnado L (2011) Homeostatic synaptic plasticity through changes in presynaptic calcium influx. J Neurosci 31:7492–7496. 10.1523/JNEUROSCI.6636-10.2011 21593333 PMC3124754

